# Chemokines and Chemokine Receptors as Therapeutic Targets in Inflammatory Bowel Disease; Pitfalls and Promise

**DOI:** 10.1093/ecco-jcc/jjx145

**Published:** 2018-02-23

**Authors:** Palak J Trivedi, David H Adams

**Affiliations:** 1 National Institute for Health Research (NIHR) Birmingham, Institute of Immunology and Immunotherapy, University of Birmingham, Birmingham, UK; 2 Liver Unit, University Hospitals Birmingham NHS Foundation Trust, Queen Elizabeth Hospital Birmingham, Birmingham, UK; 3 Department of Gastroenterology, University Hospitals Birmingham NHS Foundation Trust, Queen Elizabeth Hospital Birmingham, Birmingham, UK; 4 Centre for Rare Diseases, Institute of Translational Medicine, University of Birmingham, Birmingham, UK

**Keywords:** Eldelumab, Mongersen, Vercirnon

## Abstract

The principal targets for anti-chemokine therapy in inflammatory bowel disease (IBD) have been the receptors CCR9 and CXCR3 and their respective ligands CCL25 and CXCL10. More recently CCR6 and its ligand CCL20 have also received attention, the expression of the latter in enterocytes being manipulated through Smad7 signalling. These pathways, selected based on their fundamental role in regulating mucosal immunity, have led to the development of several therapeutic candidates that have been tested in early phase clinical trials with variable clinical efficacy. In this article, we appraise the status of chemokine-directed therapy in IBD, review recent developments, and nominate future areas for therapeutic focus.

## 1. Introduction

Effective immune surveillance involves the continuous patrolling of tissues by leukocytes able to respond to foreign antigens and thereby mount protective immune responses. This process is dependent upon the ability of leukocytes to reach tissues via the blood and then traverse vascular beds in response to local signals presented by endothelial cells. An initial capture step, classically mediated by selectins, brings a leukocyte flowing in the blood into contact with endothelium.^1^ This induces tethering or rolling, an inherently unstable transition state, that is converted into firm leukocyte arrest by chemokine-mediated triggering of leukocyte integrins to bind immunoglobulin superfamily members. Once stable adhesion is achieved, chemokines are able to provide directional migratory signals that direct leukocyte crawling within, through, and beyond the vasculature towards targets within tissue.

Chemokines encompass a group of small (8–12 kDa) heparin-binding cytokines with the ability to induce leukocyte migration.^2^ Virtually any cell type can express and secrete chemokines upon stimulation, including endothelial, epithelial, and stromal cells as well as leukocytes. Chemokines can operate as a soluble gradient to induce chemotaxis or after being immobilised on, for instance, the endothelial glycocalyx via glycosaminoglycan binding or within the extracellular matrix. This process allows multiple chemokines to be sequestered, often in multimeric forms, at sites of inflammation and prevents them from being ‘washed away’, facilitates chemokine localisation by leukocytes, and increases their affinity toward cognate receptors.^3^ Immobilised chemokines can also persist at higher local concentrations than the freely diffusible state,^2^ while enhancing the function of one another in a phenomenon referred to as chemokine cooperativity.^4^

Classification of chemokines can be based on structure and the position of conserved cysteine residues (Table 1), or functionally as to whether they play a role predominantly in the context of inflammation, or constitutively expressed under homeostatic conditions. Inflammatory chemokines are released by a wide range of cell types in response to pro-inflammatory cytokines, tissue injury, or contact with pathogens. Such chemokines are secreted early in response to pathogen recognition receptor activation on epithelial, stromal, and immune cells,^2^ to recruit the first wave of innate immune effectors including neutrophils, monocytes, natural killer (NK cells), and NKT cells. Chemokines also promote the migration of activated dendritic cells (DC) into secondary lymphoid tissues to activate adaptive immunity and the recruitment of T effectors (T_eff_) to the site of injury. Regulatory T cells (T_reg_) are recruited in a similar manner, and the balance between effector and regulatory cell recruitment will determine the outcome of the immune response. Homeostatic chemokines are constitutively expressed in lymphoid and non-lymphoid tissues where they mediate the physiological trafficking and positioning of cells involved in antigen sampling and immune surveillance. Functional distinctions are not absolute, and chemokines previously thought to be homeostatic are often induced and up-regulated at sites of chronic inflammation.^2^

**Table 1.** Chemokine-mediated recruitment in the small bowel and colon.

Site	Constitutively expressed	Increased in response to inflammation
Small bowel	CCR6 – CCL20 CCR9 – CCL25 CCR10 – CCL28 CXCR1 – CXCL5/6/8 CXCR2 – CXCL1/2/5/6 CXCR6 – CXCL16 CX3CR1 – CX3CL1	α4β7 – MAdCAM-1 (increased) VAP-1 α4β1 – VCAM-1 αLβ2 – ICAM-1 CCR2 – CCL2/7/8 CCR6 – CCL20 CCR5 – CCL3/4/5/8 CXCR3 – CXCL9/10/11 CX_3_CR_1_ – CX_3_CL_1_ (increased)
Colon	CCR5 – CCL3/4/5/8 CCR6 – CCL20 CCR10 – CCL28 CX3CR1 – CX3CL1	CCR2–CCL2/7/8 CCR3 – CCL11 (UC) CCR4 – CCL17 (murine colitis) CCR6 – CCL20 CCR9 – CCL25 (UC) CXCR1 – CXCL5/6/8 CXCR2 – CXCL1/2/5/6 CXCR3 – CXCL9/10/11

UC, ulcerative colitis.

In the gut, different intestinal sites show distinct patterns of chemokine expression, which serves to compartmentalise leukocyte recruitment, thereby regulating regional immunity. For example, under non-inflammatory conditions the chemokine CCL25 is expressed by small intestinal but not colonic epithelium, where it supports the recruitment of T cells and B cells expressing its receptor CCR9;^5^ whereas CCR10 and G protein-coupled receptor (GPR)-15 are involved in recruiting IgA^+^ plasmablasts and T cells, respectively, to the colon.^6–8^ In response to inflammation however, other chemokines can predominate; for instance interferon-induced CXCL10 can recruit CXCR3-expressing effector cells to the small bowel.^9^ Conversely in active ulcerative colitis (UC), CCL25 which was previously believed to be involved in small bowel immune homeostasis becomes detectable in the human colon, with expression levels correlating with disease activity.^10^ These findings suggest that expression of chemokines shape regional differences in immune composition along the normal intestine and determine the nature of inflammation in disease.^11–14^

## 2. CCR9 and CCL25

A greater understanding of the role of chemokines in coordinating the development and maintenance of mucosal immunity, in addition to the nature of pathological inflammation in the gut, has suggested therapeutic strategies for targeting leukocyte recruitment pathways in inflammatory bowel disease (IBD).

The chemokine CCL25 and its receptor CCR9 are essential for optimal mucosal immune development and function, with the latter being expressed by 58–97% of lymphocytes imprinted with gut-tropism.^15–18^ Activation of CCR9 by CCL25 induces pro-migratory responses, including the activation of α4β1 and α4β7 integrins to bind vascular cell adhesion molecule (VCAM)-1 and mucosal addressin cell adhesion molecule (MAdCAM)-1, respectively, on mucosal vessels.^19,20^ CCR9-CCL25 interactions shape the gut lamina propria and the development of gut cryptopatches and the intra-epithelial lymphoid compartment through recruitment of CD4 and CD8 T cells, IgA-secreting B cells, and subsets of dendritic cells.^21–26^ However, whereas lymphocytes derived from knockout animals are hampered in their ability to enter the gut in competitive homing experiments,^22^*Ccr9*^**–/–**^ mice exhibit only a mild phenotype under basal conditions with only modest reductions in numbers of intraepithelial lymphocytes (IELs),^27^ suggesting that T cells can enter the intestine through CCR9-independent as well as dependent mechanisms. This is particularly the case in the context of inflammation when other pathways including those involving CXCR3 play a more dominant role.^9^

The only known ligand for CCR9 is CCL25, expression of which is largely restricted to thymic and small bowel epithelium under normal conditions, although in response to inflammation, CCL25 is also detected in the colon and liver.^10,28^ Early growth response protein (EGR)-1 and caudal-related homeobox transcription factor (CDX) have been reported to increase CCL25 transcription in endothelial cells, but the role of conventional inflammatory stimuli such as tumour necrosis factor alpha (TNFα) or interferon (IFN)-γ remain controversial.^11,17^ Murine studies report a gradient of CCL25 expression throughout the normal gut, with highest tissue levels detected proximally in the duodenum and lowest in the ileum and very little if any detected in the non-inflamed colon.^22^ Furthermore, there appears to be differential dependence on CCR9 and CCL25 for recruitment to the lamina propria (LP) and the intestinal epithelium dependent (in part) on cell type.^9,15^

Studies of the CCR9-CCL25 axis in IBD have given different results depending on the model studied and the time course of inflammation. Neutralising antibodies to CCL25 reduced the adhesion of adoptively transferred intestinal lamina propria lymphocytes (LPL) and intraepithelial lymphocytes (IEL) to TNF-activated small intestinal post-capillary venules, but not to colonic vessels.^17^ In the SAMP1/YitFc model of murine ileitis, a significantly greater proportion of CD4^+^ and CD8^+^ lymphocytes are CCR9^+^ compared with wild-types, and histological indices of early but not late intestinal inflammation are lessened following administration of antibodies targeting the CCR9-CCL25 axis.^29^ The ileitis seen in *Rag2*-deficient mice is also attenuated in the absence of CCR9, although these findings conflict with the *Tnf*Δ^*ARE*^ model of IBD, wherein CCR9 deficiency is associated with a reduction in gut T_reg_ and persistent intestinal inflammation.^30–32^ Consistent with the latter, CCR9 can be expressed by subsets of T_reg_ and immunosuppressive DC populations.^25,30,31,33^ Indeed, following adoptive transfer of CD4^+^ T-cells, *Ccr9*^*-/*-^*Rag2*^*-/*-^ mice develop small bowel inflammation, suggesting that non-T cell CCR9^+^ populations are partially responsible for maintaining tolerance in this model. Furthering the argument for an immune modulatory role of CCR9 cells, Wurbel *et al*. reported that CCR9-CCL25 interactions are critical for protective responses against large intestinal inflammation in acute and chronic models of dextran sulphate sodium (DSS)-induced colitis.^34,35^

There are several potential explanations for these different findings. Imprinting of CCR9 is retinoic acid dependent, and in the presence of transforming growth factor (TGF)-β is associated with induction of FoxP3. Indeed, CD4^+^ T_reg_ preferentially express CCR9 when compared with effector counterparts in the *Tnf*Δ^*ARE*^ model, and CCR9^+^ CD8^+^ subsets can inhibit of proliferation of CD4^+^ T-cells *in vitro*. Wermers *et al*. report highly concordant expression of CCR9, FoxP3, and CD103 in mice, suggesting that CCR9 is required for T cell-mediated regulation of chronic ileitis.^30^ Moreover, CCR9-CCL25 interactions are critical for establishing early thymocyte colonisation and selective maturation of T cells that are tolerant to self-antigens (hence the synonym for CCL25: thymic expressed chemokine; TECK).^26^ However, clinical studies illustrate increased numbers of CCR9^+^ T cells in blood from individuals with small bowel inflammation;^36^ and CCR9^+^ T cells isolated from mesenteric lymph nodes (MLN) of patients with Crohn’s disease are more activated and show enhanced IFN-γ and interleukin (IL)-17 secretion when compared with lymphocytes extracted from normal MLN.^36,37^

Collectively, these findings imply that although congenital absence has potentially harmful consequences, targeted therapy against CCR9 or CCL25 may be beneficial in the prevention or treatment of small bowel inflammation. This hypothesis is supported by clinical trial data in which clinical and endoscopic indicators of small-intestinal Crohn’s disease improved following treatment with the CCR9-antagonist Vercirnon.^38^

Whereas a role for lymphocyte CCR9 is accepted in the small bowel, controversy has existed over the roles of CCR9 and CCL25 in the colon.^37,39^ Murine studies suggest that under non-inflammatory conditions, *Ccl25* gene expression is restricted to the small intestine in uninjured wild-type mice^9,24,40^ and that is also the case in some IBD models, including the Samp1/YitFc model of ileitis,^29^ the *TnfΔ*^*ARE*^ model of small bowel Crohn’s disease,^30^ and spontaneous ileitis in *Rag2*^*-/*-^ mice.^31^ Studies in pigs again suggest differential expression along the small intestine which is dependent on the microbiota.^41^ Nevertheless, colonic CCL25 transcripts have been reported in spontaneous murine models of colitis,^42^ as well as colonic inflammation induced by dextran sulphate sodium^34,35^ and oxazolone.^43^

In humans there has been even more variability in findings. In some studies, fewer than 10% of cultured T cells isolated from colonic lymphoid tissue expressed CCR9 irrespective of inflammatory status, and lower frequencies of circulating CD4^+^ T cells expressed CCR9 in patients with colonic versus small bowel Crohn’s disease.^39^ Consistent with this, northern blotting was able to detect CCR9 mRNA in small bowel but not colonic tissue.^16^ In other studies, increased CCR9 expression gauged by median fluorescence intensity (MFI) has been reported on subsets of CD3^+^ T cells and CD14^+^ monocytes in the blood of a small number of UC patients.^44,45^ IL-17^+^ CCR9^+^ γΔ T cells have also been implicated in the mediation of extraintestinal inflammation in humans, and are increased in the blood of patients with active UC.^46,47^ Recent studies from our group and others have extended upon these observations, to confirm elevated serum levels of CCL25 in patients with UC^48^ and, most significantly, a strong positive correlation between *CCL25* gene expression in the colonic mucosa and both the Mayo endoscopic sub-score and mucosal TNFα levels in ulcerative colitis patients.^10^ This was validated by CCL25 protein expression in the inflamed colon and a high frequency of CCR9^+^ colon-infiltrating effector T cells.^10,42^ Such findings may contribute to the increased colonic cancer risk in colitis which relates to inflammatory burden, given the ability of CCR9-CCL25 interactions to mediate colonic tumour growth, invasion, and metastasis.^49^

### 2.1. Therapeutic targeting of CCR9/CCL25

The biological role of the CCR9-CCL25 axis and the evidence implicating CCR9, and CCL25 in gut inflammation in both human and murine IBD, suggest this is an appropriate pathway to target therapeutically.^10,29,39,50,51^ However, the involvement of CCR9 and CCL25 in immune regulation and tolerance raises valid concerns that under some circumstances inhibition could be detrimental. Studies of immunoblockade of CCR9 and CCL25 in the Samp1/Yit model, showing an effect on early but not late inflammation, suggest that timing of intervention may also be important, with the possibility of a role in preventing or inducing rather than maintaining remission.^29^ The only orally bioavailable CCR9 antagonist, CCX282-B (Chemocentryx, USA)^52^ is a potent inhibitor of CCR9^+^ T cell-mediated chemotaxis *in vitro*, and results in near complete protection against ileitis in the *TnfΔ*^*ARE*^ model.^34^ Additionally CCX282-B attenuated colitis in oxazolone-treated animals.^43^

The encouraging results from preclinical models have thus led to clinical study in IBD. The PROTECT-1 phase IIb trial randomly allocated patients with Crohn’s disease to placebo or one of three treatment dosages, organised into: an induction phase (induction of clinical response at Weeks 8 and 12); an active, open-label study phase (4 weeks) in which all eligible participants received CCX282-B at 250 mg twice daily; and a maintenance period in which patients who showed clinical response during the active phase were re-randomised to receive placebo or CCX282-B at a dose of 250 mg twice daily. The induction phase of PROTECT-1 failed to attain its primary endpoint of a significant reduction in the CD Activity Index (CDAI) of 70 points at Week 8 of treatment, although a greater number of patients on the 500 mg daily dose regimen achieved clinical response compared with placebo (Table 2). The fact that response rates were similar between placebo and the 250 mg treatment groups suggests this dose may be suboptimal, although 41% of subjects on CCX282-B were in corticosteroid-free remission compared with 28% on placebo (*P* = 0.041). The active treatment phase of PROTECT-I gave all patients an opportunity to receive active treatment, following which patients who showed a clinical response were re-randomised to receive placebo or CCX282-B (250 mg twice daily) as maintenance therapy. Remission was achieved in 47% of patients on CCX282-B treatment compared with 31% of those on placebo (*P* = 0.012), together with a reduction in median overall CDAI.

**Table 2.** Clinical trials of anti-CCR9 therapy in IBD.

Compound	Entry criteria	Cohort size	Organisation	Primary endpoint	Potential expedients
A) Phase II (PROTECT)					
CCX282-B (oral)	**Inclusion:** Moderate to severe Crohn’s disease - CDAI 250450 - Fasting CRP > 7.5 mg/L **Exclusion:** - Fluctuating dosages of immunosuppression 4 weeks^a^ - > 20 mg prednisolone (or equivalent) 4 w previously^a^ - Anti-TNFα or anti-α4 integrin treatment 12 w previously^a^	- Placebo twice daily (*n* = 144) - 250 mg CCX282-B once daily (*n* = 98) - 250 mg CCX282-B twice daily (*n* = 96) - 500 mg CCX282-B once daily (*n* = 97)	**Induction phase; 12 w** - Measuring CR at 8 and 12 w **Active (open-label) period; 4 w** - Eligible participants received CCX282-B (250 mg twice daily) **Maintenance period; 36 w** - Subjects with clinical response following active period re-randomised to receive placebo or CCX282-B (250 mg twice daily)	**Induction period** CDAI to ≤ 70 by 8 w - Placebo: 49% - 250 mg o.d.: 52% - 250 mg b.d.: 48% - 500 mg: 60% *P* = N.S.; all comparisons **Maintenance period** Sustained CDAI ≤.70 - Placebo: 31% - 250 mg b.d.: 47% *P* = 0.012	**CDAI ≤ 70 by 12 w** - Placebo vs 500 mg: 47% vs 61%; *P* = 0.039 **CDAI decrease by ≥ 100 points by 12 w** - Placebo vs 500 mg: 40% vs 55%; *P* = 0.029 **Mean change in CDEIS at 12 w** - Placebo: - 3.0 - 250 mg o.d.: - 8.7 - 250 mg b.d.: 2.0 - 500 mg: - 10.8 *P* = 0.049 for 500 mg vs placebo
B) Phase III (SHIELD-1)^54^					
CCX282-B (oral): - Vercirnon	**Inclusion:** Moderate to severe Crohn’s disease - CDAI 220450 - Active inflammation^b^ - Inadequate response to immunosuppression **Exclusion:** - Fluctuating dosages of immunosuppression^a^ - > 20 mg prednisolone (or equivalent) - No concurrent dependence on anti-TNFα therapy during trial - EC, abdominal or pelvic fistulas with abscess	- Placebo once daily (*n* = 203) - 500 mg CCX282-B once daily (*n* = 203) - 500 mg CCX282-B twice daily (*n* = 202)	**Induction phase only**	**CDAI dec. ≥100 by 12 w** - Placebo: 25% - 500 mg o.d.: 27% - 500 mg b.d.: 27% *P* = N.S.; all comparisons	**CDAI dec. ≥ 100 by 12 w in patients with colitis** - Placebo: 13% - 500 mg o.d.: 25% - 500 mg b.d.: 29% *p* < 0.05; for 500 mg b.d. vs placebo
C) Phase II^55^,^56^					
CCR9-targeted leukapheresis	**Inclusion:** UC; moderate to severe activity - Mayo score 6–11 - Prednisolone ≤ 20 mg daily (stable dose at least 2 w) - Naïve to anti-TNFα antibodies	- Placebo (*n* = 9) - Treatment (*n* = 14)	**Alternate day leukapheresis** **(5 sessions; 10 days)**	**Reduction of CCR9** ^**+**^ **HLA-DR** ^**hi**^ **monocytes** - 11% to 12% (*P* = 0.469) vs 14% to 10% (*P* = 0.039); placebo vs treatment groups, respectively	**Dec. in overall Mayo score:** 8.0 to 6.3 (*P* = 0.125) vs 8.8 to 5.7 (*P* = 0.016); placebo vs treatment groups, respectively

IBD, inflammatory bowel disease; CRP, C-reactive protein; EC, enterocutaneous; TNF, tumour necrosis factor; CDAI,Crohn’s Disease Activity Index; CDEIS, Crohn’s Disease Endoscopic Index of Severity; w, weeks; m, months; UC, ulcerative colitis; N.S., not significant; dec., decrease; o.d., once daily; b.d., twice daily.

^a^Before randomisation.

^b^By endoscopic assessment or a CRP ≥.3 mg/L or faecal calprotectin > 200 µg/g in stool.

Following the results of the phase IIb study, CCX282-B was reformulated and renamed (initially GSK1605786 Formulation A, and later Vercirnon) and studied in a phase III double-blind randomised placebo-controlled trial. SHIELD-1 was conducted over 162 centres in 23 countries between 2010 and 2013, and included patients with a CDAI of 220–450, evidence of active Crohn’s disease endoscopically, or with elevated inflammatory markers, and if they had failed corticosteroid or immunosuppressive therapy.^54^ No significant difference in remission rates (defined as a reduction in CDAI of ≥ 100 points by Week 12) was observed between either of the treatment arms compared with placebo (Table 2).

The reasons for the discrepancy in outcome between the Phase IIb PROTECT-1 study and the Phase III SHIELD-1 study are unclear but could reflect differences in the patients recruited; previous anti-TNFα treatment was reported in 69% of patients comprising SHIELD-1 versus 26% in PROTECT-1. Another plausible reason for failure in the phase III Vercirnon study may stem from differences in clinical trial design. For instance, PROTECT-I comprised a 12-week placebo-controlled lead-in followed by 4 weeks of open-label treatment.

It is also possible that 12 weeks is insufficient time for the drug to deplete the lamina propria of effector populations, because previously recruited cells may be retained for several weeks before they die *in situ*. This would be consistent with the positive effects on maintenance of remission reported in PROTECT-1.^51^ Although results from the maintenance phase of the SHIELD programme have not been published, the aim of SHIELD-4—a randomised double-blind induction study for patients with moderate-to-severe Crohn’s disease—was to treat patients with one of two dose regimens of Vercirnon, and enrol clinical responders to a Phase III maintenance clinical trial (SHIELD-2).^55^ The primary endpoint of the trial was the proportion of patients with a Crohn’s disease activity index (CDAI) ≥ 100-point response (100-point decrease in CDAI score) at Week 12. The secondary endpoint was the proportion of patients in clinical remission (CDAI < 150) at Week 12. Adult patients with a baseline CDAI of 220 to 450, C-reactive protein (CRP) ≥ 3 mg/L or faecal calprotectin > 200 μg/g were randomised to receive either 500 mg four times daily (q.d.s.) or 500 mg twice daily (b.d.) Vercirnon for 12 weeks. Baseline CDAI was 323 (± 56) with a range of 220–450. In those who completed the trial before premature study termination (*n* = 118), the CDAI ≥ 100-point response at Week 12 was 56% and 69% in the 500-mg q.d.s. and in the 500-mg b.d. groups, respectively, similar to the PROTECT-1 data. Within the same observed population, rates of remission (defined as CDAI < 150) at Week 12, were 26% and 36%, respectively. To the authors’ knowledge, no further data relating to the SHIELD-2 maintenance study are available.

The failure to reach primary endpoints in the phase III clinical trial has delayed further development of anti-CCR9 therapy in IBD. However, pre-specified subgroup analysis in SHIELD-1 did show a higher clinical response for Vercirnon treatment compared with placebo, specifically in patients with colonic disease: CDAI response at Week 12 of 25.4%, 28.8%, and 13% in the once daily, twice daily, and placebo groups, respectively.^57^ Although the investigators state a lower placebo response rate as one factor that could explain this observation, it is also possible that this was a real effect consistent with the fact that CCR9^+^ T cell frequencies do not correlate with inflammatory activity in the small bowel, whereas they correlate strongly with disease activity in the colon.^10^ The current evidence therefore indicates that a randomised placebo-controlled trial of Vercirnon, specifically for patients with ulcerative colitis, is justified.

In addition to circulating gut homing T cells, subsets of pro-inflammatory monocytes also express high levels of CCR9,^44^ and their frequencies in the circulation correlate with clinical activity in some IBD patients. A recently published study reports the successful treatment of a patient with refractory UC, by depleting CCR9 cells from the blood using leukapheresis. Clinical and endoscopic remission was associated with a greater reduction in CCR9 expression on circulating T cells and monocytes.^45^ This led to a subsequent double-blind placebo-controlled trial of leukapheresis to deplete CCR9^+^ monocytes in ulcerative colitis, which was associated with improvement in the endoscopic Mayo score compared with placebo (Table 2).^56^

## 3. CXCR3 and CXCL10

CCL25 and CCR9 drive gut specific leukocyte recruitment under homeostatic and inflammatory conditions, but in the context of IBD their role can be overshadowed by inflammatory chemokines.^11,12^ Human LPL and IEL express the chemokine receptors CCR2, CCR5, and CXCR3 that play an important role during leukocyte recruitment to the inflamed intestine.^11^ Under normal circumstances the colonic epithelium expresses low levels of the chemokine ligands for CXCR3 (namely CXCL9, CXCL10, and CXCL11). However, the expression of one of these, CXCL10, which is an IFN-γ inducible chemokine, increases markedly in colitis.^57,58^ CXCR3 is expressed at high levels on tissue-infiltrating, activated T cells in a number of inflammatory disorders, and mucosal biopsies taken from patients with active UC and Crohn’s disease demonstrate increased frequencies of CXCR3^+^ cells associated with strong expression of CXCL10.^59–61^ Of note, the latter has been implicated in the induction of T_h_1 responses across inductive sites such as MLNs, as well as the promotion of effector cell recruitment to inflamed gut tissue. Zhao *et al*. have also demonstrated that the CXCL10/CXCR3 axis mediates monocyte activation and drives tissue inflammation by innate immunity distinct from the conventional paradigm as a regulator of immune cell recruitment.^62^ In contrast to CXCL9 and CXCL11, CXCL10 alone was necessary and sufficient for IFN-γ primed monocytes to promote pro-inflammatory cytokine release, indicating a non-redundant role of CXCL10 in inflammation. Moreover, CXCL10 signalling was able to synergise with lipopolysaccharide to potentiate much greater amounts IL-12 and IL-23 release by monocytes.

A therapeutic role targeting CXCR3 is supported by studies in the *Il-10*^*–*^/^*–*^ model of IBD. Frequencies of CXCR3^+^ cells increase in mesenteric lymph nodes and Peyer’s patches associated with T_h_1-mediated injury, and inhibition of CXCL10 attenuates the rate and intensity of colitis and intestinal inflammation.^63,64^ CXCR3-deficient mice also develop a much milder colitis in response to dextran sulphate sodium. This is associated not only with reduced inflammation but also changes in epithelial cell proliferation, suggesting direct effects of CXCR3 chemokines on epithelial cells in the gut.^65,66^

### 3.1. Clinical trials targeting CXCR3/CXCL10

Eldelumab (BMS-936557, previously named MDX-1100^67^) is a fully human monoclonal antibody to CXCL10 which has been investigated for the treatment of moderate to severe UC in a phase IIa multicentre, double-blind randomised placebo-controlled trial.^68^ Despite a trend toward increased clinical response at Day 57, as evidenced by clinical remission and mucosal healing, there were no significant differences in the primary or secondary endpoints between treatment versus placebo groups (Table 3). However, the study yielded a notable exposure-response relationship, with Eldelumab clinical response and mucosal healing rates being greatest in patients with the highest trough concentrations.^68^ We have observed similar outcomes in a trial of NI-0801, a monoclonal antibody against CXCL10, in patients with the immune-mediated liver disease primary biliary cholangitis (PBC), which suggests that the high production rate of CXCL10 by inflamed tissues makes it difficult to achieve therapeutic drug levels and sustained neutralisation of the chemokine *in vivo* (K. de Graaf *et al;* submitted).

**Table 3.** Clinical trials targeting CXCR3/CXCL10 in IBD.

Compound	Entry criteria	Cohort size and organisation	Primary endpoint	Potential expedients
A) Phase IIa^68^				
BMS-936557 (IV)	**Inclusion:** UC; active flare despite 5-ASA or immunosuppression^a^ - Mayo score 6–10 - Mayo endoscopic sub-score ≥ 2 **Exclusion:** - Fulminant UC - Anti-TNFα therapy in preceding 8 w	- Placebo (*n* = 54) - BMS-936557 10 mg/kg (*n* = 55) **Induction period;** 8 w	**Dec. in Mayo score ≥ 3 at 57 d + dec. in rectal bleed score ≥ 1 or absolute bleed score ≤ 1** - Placebo 35% - 10 mg/kg: 53% *P* = 0.083	**Attaining primary endpoint by dose-response (drug trough drug values**) - *Placebo*: 37% - 26–79 µg/mL: 53% - 79–105 µg/mL: 63% - 108–235 µg/mL: 88% *Mucosal healing* - Placebo: 35% - 26–79 µg/mL: 29% - 79–105 µg/mL: 44% - 108–235 µg/mL: 69%
B) Phase IIb^69^				
BMS-936557 (IV) - Eldelumab	**Inclusion:** UC; moderate to severe activity^a^ - Mayo score ≥ 6 - Mayo endoscopic subscore ≥ 2 - Inadequate response to existing medical therapy; any type **Exclusion:** - As above	- Placebo (*n* = 83) - Eldelumab 15 mg/kg (*n* = 84) - Eldelumab 25 mg/kg (*n* = 85) **Induction period;** 11 w	**Mayo score < 2 by 11 w + no individual component > 1** - Placebo: 9.6% vs - 15 mg/kg: 13.1%; *P* = 0.515) - 25 mg/kg: 17.6%; *P* = 0.158)	**Subgroup analysis of primary endpoint and mucosal healing greater compared with placebo in:** (A) anti-TNFα naïve; and (B) immunomodulator-exposed subgroups
C) Phase IIa^70^				
BMS-936557 (IV) - Eldelumab	**Inclusion:** Moderate to severe Crohn’s disease - CDAI 220 to 450 - CRP ≥ 5 mg/L, or - Faecal calprotectin > 250 µg/g, or - SES-CD of 2 to 3 - Insufficient response/intolerance to prednisolone (≥ 40 mg/d for 2 w (or equivalent) or immunomodulators **Exclusion:** - Penetrating disease or fibrotic stenosis - Surgery within 6 m of screening - Anti-TNFα therapy in preceding 8 w	- Placebo (*n* = 40) - Eldelumab 10 mg/kg (*n* = 40) - Eldelumab 20 mg/kg (*n* = 41)	**1) Steady-state plasma concentration for induction**- No statistically significant exposure-remission relationship with Eldelumab at 11 w OR: 1.06 (90% CI 0.96–1.18) **2) Efficacy: CDAI < 150 by 11 w;** treatment differences - 10 mg/kg vs placebo: 9% - 20 mg/kg vs placebo: 13%	**Composite endpoint analysis showed increased response rate for both doses vs placebo**

5-ASA, 5-aminosalicylic acid; CDAI, Crohn’s Disease Activity Index; CI, confidence interval; d, day; dec., decrease; IV, intravenous; m, month; SES-CD, simplified endoscopic score of Crohn’s disease; TNF, tumour necrosis factor; OR, odds ratio; UC, ulcerative colitis; w, week, IBD, inflammatory bowel disease; CRP, C-reactive protein.

^a^2 w before drug administration and of ≥ 6 m duration.

Based on the phase IIa data, a 100-µg/mL trough value was considered the ‘target for efficacy’ in a phase IIb dose-ranging induction study in UC (Table 3).^69^ The proportion of patients achieving clinical remission by Week 11 (primary endpoint) was no different between placebo versus either the Eldelumab 15 mg/kg or the Eldelumab 25 mg/kg group. This was despite 79% and 97% of patients in the treatment groups maintaining a trough plasma concentration of 100 µg/mL, respectively. Although a numerically higher proportion of patients in the Eldelumab treatment arms achieved clinical response, rates of mucosal healing were similar when compared with placebo. In subgroup analysis, greater efficacy was observed in Eldelumab-treated patients who were anti-TNFα naïve,^69^ and for the 25 mg/kg subgroup who were receiving concurrent immunomodulators.

Results from a phase IIa trial in Crohn’s disease were also recently published.^70^ The primary objective was to demonstrate dose response; no exposure-remission relationship was seen with Eldelumab, although a trend towards clinical and endoscopic efficacy was observed. There was no statistically significant relationship between trough drug levels and clinical remission by Week 11, suggesting that at the doses of 10 and 20 mg/kg studied, clinical efficacy had been maximised.^70^ Moreover, there were no significant differences in the rate of remission or CDAI response rates between either the 10 mg/kg or 20 mg/kg treatment groups versus placebo (Table 3). As with previous clinical trials in IBD, clinical response was more pronounced for treatment subgroups who were anti-TNFα naïve or receiving concurrent immunomodulatory treatment.^70^

This was one of the first placebo-controlled, prospective studies with central endoscopy reading for patients with moderate to severe Crohn’s disease. This follows lessons from the earlier UC trial of Eldelumab as well as others, in which high but variable mucosal response rates were seen with placebo.^69^ Treatment with both Eldelumab doses resulted in mean reductions in the endoscopic degree of inflammatory activity measured by the simplified endoscopic score in Crohn’s disease (SES-CD): 3.44, –3.57, and –0.94, for 10 mg/kg, 20 mg/kg, and placebo groups, respectively).^70^ Unlike with clinical response, endoscopic activity following Eldelumab did not differ in those patients with previous exposure to biologic therapy,^70^ neither did this vary according to baseline severity of endoscopic disease. Although the trial was not powered to test efficacy endpoints, given the lack of agreement between clinical and endoscopic findings a composite *post hoc* endpoint analysis was conducted. Therein, response was determined if ≥ 30% improvement in clinical symptoms occurred together with either: (i) a 3-point decrease in SES-CD score; or (ii) an absolute SES-CD score of zero. This did show significant treatment differences between Eldelumab and placebo for both the biologic naïve and biologic failure subgroups, albeit with very low placebo response rates.

The above studies all indicate that inhibition of the CXCR3-CXCL10 pathway appears to be safe but fails to demonstrate overall efficacy. However, the suggestion of responses in specific patient populations are encouraging. It is possible that IBD results in the activation of several inflammatory pathways and thus inhibition of CXCR3 alone is insufficient to induce clinical responses. However further attempts to define patients based on clinical or endoscopic features, or perhaps more pertinent immunophenotypes, might allow treatment to be targeted to patient subgroups more likely to experience therapeutic benefit.

## 4. CCR6 and CCL20

The ability of intestinal epithelial cells to secrete cytokines and chemokines in response to commensal bacteria, pathogens, or injury allows them to play an active role in shaping the nature of the local immune response.^71^ IL-17-secreting innate lymphoid cells and T_h_17 CD4 T cells are abundant in the gut, and the epithelium-secreted chemokine CCL20 plays a major part in attracting IL-17-secreting cells that express its sole receptor CCR6.^72^ Although CCL20 is secreted by the epithelium in response to inflammation, it is also constitutively expressed by the follicle-associated epithelium overlying Peyer’s patches and isolated lymphoid follicles through which it contributes to the homeostatic development and maintenance of the mucosal immune system. In *Ccr6* knockout mice, there is a failure of full development of mucosal inductive sites leading to decreased IgA production to oral antigens. Thus, CCL20 functions as both an inflammatory and a homeostatic chemokine.^73^

Following epithelial damage, infection, or changes in the microbiota, there is a shift in local mucosal immune responses away from regulation and towards pro-inflammatory Type-1 or Type-17 responses, driven in part by increased production of CCL20 from inflamed epithelium.^12,74–78^ A role for CCL20 in IBD pathogenesis is also supported by the findings of genome association and gene expression studies,^79,80^ and the fact that intestinal CCL20 mRNA expression is up-regulated several-fold during active inflammation,^77,78^ enhanced through stimulation of toll-like receptor (TLR)-1 and TLR3.^77,81^ Murine models have also shown that T_h_17-cells can be actively redirected from the blood to the small bowel in response to CCL20,^82^ and several studies report that IL-17A in turn up-regulates CCL20 release from the intestinal epithelium, resulting in a positive feedback circuit that drives ongoing inflammation.^82^ Furthermore, neutralisation of CCL20 reduces T cell recruitment and attenuates colitis in the 2,4,6-trinitrobenzene sulphonic acid (TNBS)-induced murine model.^83^

Persistent intestinal inflammation requires not only local pro-inflammatory signals such as TNFα that induces CCL20 secretion by epithelial cells,^10^ but also the presence of induced or constitutive defects in counter-regulatory mechanisms that allow inflammation to develop unchecked.^84^ One such mechanism involves TGF-β1, levels of which are reduced in CD and UC due to increased expression of the intracellular protein Smad7.^85^ TGF-β1 activity can be restored in isolates of lamina propria mucosal cells by treatment with an anti-sense oligonucleotide to Smad7 termed Mongersen (previously GED0301), the administration of which attenuates colitis in the TNBS mouse model.^86^ Smad7-transgenic animals do not develop an IBD phenotype under normal circumstances but exhibit more severe colitis in response to DSS than wild-types.^87^ Moreover, Smad7-overexpressing CD4^+^ T-cells induce severe colitis that is resistant to T_reg_-mediated rescue when transferred to immunodeficient mice.^88^ Marafini and colleagues have recently shown that CCL20 expression by colonic epithelial cells was inhibited by TGF-β1, but that this was overridden in epithelial cells that over-expressed Smad7.^89^ Moreover, Mongersen administered directly to the luminal side of mucosa down-regulated Smad7 in a dose-dependent manner, which correlated with the reduction in colonic CCL20 expression.^89^

### 4.1. Targeting Smad7 and the impact on CCL20 expression

In a small open-label and dose-finding Phase I study in Crohn’s disease,^90,91^ treatment with Mongersen was associated with a reduction in CDAI of > 70 points within 8 days across all three tested doses (40 mg, 80 mg, 160 mg; *n* = five participants per group). These results were swiftly followed by efficacy evaluation in a Phase II, multicentre, double-blind, placebo-controlled trial.^92^ A total of 166 active, steroid-dependent or steroid-resistant Crohn’s disease patients were assigned to receive one of three doses of Mongersen (10, 40, or 160 mg per day) or placebo daily for 2 weeks. The primary endpoint was the proportion of individuals who reached clinical remission at Day 15 (CDAI < 150) maintained for at least 2 weeks. Patients receiving the highest doses of Mongersen experienced significantly higher rates of remission than those treated with 10 mg or placebo (Table 4). Moreover, at the end of follow-up on Day 84, the percentage of patients who entered glucocorticoid-free remission was significantly greater in the 160 mg group versus placebo. Colonic Smad7 expression and CCL20 levels were decreased in treated patients, and serum values were also reduced in those achieving clinical response as early as Day 15 of treatment,^89^ suggesting that circulating CCL20 may be a useful biomarker to monitor early response, efficacy, and dose titration.

**Table 4.** Summary data from the Mongersen Phase II clinical trial in Crohn’s disease (orally administered).

Phase II ^92^			
Entry criteria	Cohort size and organisation	Primary endpoint	Additional expedients
**Inclusion:** - Moderate to severe Crohn’s disease (CDAI 220400) - Disease located to the terminal ileum of right colon - Steroid-dependent / refractory disease **Exclusion:** - Disease affecting the upper GI tract, proximal small intestine, transverse colon, or left colon. - Stricturing disease - Penetrating disease - Extraintestinal disease - Anti-TNFα therapy in preceding 90 d^a^	- Placebo (*n* = 42) - Mongersen 10 mg (*n* = 41) - Mongersen 40 mg (*n* = 40) - Mongersen 160 mg (*n* = 43) **Daily treatment for 2 w** - **Clinical evaluation at 15 d, 28 d, and 84** d	**CDAI < 150 at 15 d, and sustained until 28 d** - Placebo: 10% - 10 mg: 12% - 40 mg: 55% - 160 mg: 65% *P* < 0.001 for 40 mg or 160 mg groups vs either 10 mg or placebo groups	**100-point CDAI response at 15d** - Placebo: 26% - 10 mg: 22% - 40 mg: 45% - 160 mg: 65% *P* < 0.05 for 40 mg or 160 mg groups vs either 10 mg or placebo groups **100-point CDAI response at 28d** - Placebo: 17% - 10 mg: 37% - 40 mg: 58% - 160 mg: 72% *P* ≤ 0.001 for 40 mg or 160 mg groups vs either 10 mg or placebo groups *P* < 0.05 for 10 mg vs placebo groups

CDAI, Crohn’s disease activity index; d, days; TNF, tumour necrosis factor; w, weeks; GI, gastrointestinal.

^a^Before the date of enrolment. However, patients with a worsening of disease (increase in CDAI ≥ 70 points) after 2 weeks into the trial were eligible for steroid, immunomodulator or anti-TNFα rescue.

A *post hoc* subgroup analysis of the Mongersen phase II data divided the cohort according to baseline disease duration (cut-point of 5 years), CDAI (cut-point of 260), and CRP level (cut-point of 3 mg/L).^93^ The authors found that clinical remission rates (CDAI < 150) at Weeks 2, 4, and 12 were significantly higher with Mongersen 160 mg/day and 40 mg/day compared with placebo for each disease duration and CRP subgroup. Furthermore, the rate of remission was significantly greater with Mongersen 40 mg/day compared with placebo at Weeks 2, 4, and 12 in the subgroup of patients with CDAI ≤ 260. The authors concluded that disease duration and baseline CRP did not appear to significantly affect drug efficacy. However, endoscopic indices of disease severity were not evaluated, and data from other trials suggest that baseline CDAI does not correlate with endoscopic remission.^94^ A phase III trial of Mongersen is ongoing, and these studies have also encouraged interest in direct anti-CCL20 therapy, which is now being explored by GSK in a Phase I clinical trial of an anti-CCL20 antibody GSK3050002 (NCT01984047).

## 5. Critical Appraisal and Future Directions

Although it is clear that chemokines and their receptors are critical in the development of the mucosal immune system and in orchestrating inflammatory responses to tissue injury and infection, only a few have been explored as targets for IBD.^95^ This may partly reflect current therapeutic focus within the field in targeting endothelial adhesion molecules, with agents such as vedolizumab. Additionally, concerns surround safety of targeting chemokine receptors in light of their widespread expression on a broad range of cell types.^96^ For instance, a potential ligand for GPR15 (GPR15L) was recently characterised as being developmentally determined, only modestly affected by inflammation or the presence of microbiota, and constitutively expression in the colon.^97^ These findings called into question the therapeutic implications of targeting GPR15, given its role in mediating colitis in T cell-dependent murine models, and high expression levels on T_h_2 cells in the lamina propria of patients with UC.^7,8^ However, GPR15 is critical for regulating the migration of FoxP3 T_reg_ to the large intestine, and is also expressed on vascular endothelium at similar levels to T cells, where it binds to thrombomodulin, is cytoprotective, and mediates critical angiogenic functions *in vivo.*^98^ In a similar vein, maraviroc, which was developed as a therapy for HIV infection, is a safe and effective inhibitor of CCR5.^99^ The latter is also expressed at high frequencies by gut infiltrating lymphocytes and monocytes in IBD,^100^ suggesting it could be a promising target. However, its distribution on a broad range of leukocytes including activated, highly proliferative immunosuppressive T_reg_ populations in the gut, suggest that inhibition may also disrupt critical regulatory pathways. Other potentials implicated in IBD pathogenesis include CCL17 and its only known receptor CCR4. CCL17 is increased in the inflamed mucosa of murine colitis,^101,102^ and CCL17-deficient animals are protected from colitis induced by DSS,^103^ although the ability of mice lacking CCR4^+^ T-cells to develop florid colitis suggests contribution by CCR4^+^ innate effector cell types.

There is also a strong rationale to target gut-tropic chemokines in patients with extraintestinal manifestations of IBD. Of particularly relevance is primary sclerosing cholangitis (PSC),^12^ a progressive biliary disease that is associated with IBD in 70–80% of cases and which affects ~8% of patients with IBD, particularly colitis.^104,105^ Under normal circumstances, expression of CCL25 and MAdCAM-1 are absent from the liver, but in PSC both are detectable on hepatic endothelium^28,106^ and support the aberrant recruitment of α4β7^+^ CCR9^+^ effector lymphocytes from the gut.^10,28,106^ These effector cells can then exploit CCR6 to localise to biliary epithelium, where they drive injury upon reactivation within the liver.

Despite advances in our understanding, strategies that target chemokine receptors do provide unique challenges,^96^ and have not yet achieved the same level of efficacy as those directed toward integrins or endothelial adhesion molecules. Although safe and reasonably well tolerated in most studies (Table 5), it has proved difficult to deliver consistent inhibition with antibodies, which may reflect the high levels of chemokines secreted by inflamed tissues or their sequestration on the endothelial glycocalyx. Indeed, targeting of chemokine-receptor interactions may be less efficacious than therapies which inhibit binding of chemokines to glycosaminoglycans *in vivo.*^107^

**Table 5.** Safety and tolerability profiles of existing chemokine-directed agents.

Agent	Commentary
CCX282-B (oral); Vercirnon	- No specific safety concerns in the Phase II study^38^ - In the Phase III trial,^54^ gastrointestinal adverse events were observed at an increased frequency in the Vercirnon treatment groups: 30%, 37%, and 48% for placebo, Vercirnon once daily, and Vercirnon twice daily respectively (*P* < 0.001, 500 mg twice daily vs placebo), with the most common AEs being abdominal pain, Crohn’s disease worsening, nausea, and dyspepsia
BMS-936557 (IV); Eldelumab	Ulcerative colitis phase II study^69^ - Infusion reactions occurred in 19%, 14%, and 5% in the 25 mg/kg, 15 mg/kg, and placebo groups, respectively. None with an acute infusion reactions exhibited anti-Eldelumab antibodies - Higher proportion of infectious complications in the 25 mg/kg group patients (26%) vs 15 mg/kg (17.9%) and placebo (18%) groups. The most common type of infection was nasopharyngitis (10.6%, 3.6%, and 3.6%, respectively) Crohn’s disease phase II study^70^ - Infusion reactions in 10% and 27% of patients receiving 10 mg/kg and 20 mg/kg arms; in three cases (all in the 20-kg arm), infusion reactions were considered serious
Mongersen (oral)	- No serious concerns in the Phase II trial^92^ - Theoretical risk of fibrotic stricturing disease, attributable to the role of TGF-β1 in collagen synthesis, extracellular matrix dynamics, and fibrogenesis. However, no patient developed small bowel strictures in a 6-month phase I study^91^

AE, adverse event.

Additional limitations, relevant to cytokine-directed therapies more broadly, include acceptance that therapeutic drug monitoring in peripheral blood may not necessarily reflect levels in mucosal tissue. This was highlighted by the ATLAS study, which revealed no correlation between anti-TNFα values in matched specimens of serum versus mucosal biopsies from patients with active IBD.^108^ Moreover, the ratio of anti-TNF to TNF in tissue was greatest in non-inflamed areas and lowest in the most severely afflicted regions. These observations have not been conclusively validated,^109^ but the ATLAS investigators propose that local, progressive inflammation characterised by high tissue levels of TNFα serves as a ‘sink’ for anti-TNFα. It is plausible that the reason why some therapies fail in clinic is thus because of potency issues, wherein high levels of compound are required to ‘draw out’ chemokines from out of tissue compartments.

In future, it will be important to look at new ways of modelling pharmacokinetics for such antibodies, to ensure that trials are not failing due to suboptimal dosing.^52^,^53^ Furthermore, the large numbers of different players secreted in the context of disease suggest a degree of redundancy in inflammation-induced pathways, which could potentially reduce the efficacy of therapy targeted at single chemokines or receptors. This suggests that targeting chemokines that play a specific role in the gut or in the recruitment of particular, critical immune subsets may be more effective than targeting receptors involved more broadly during an inflammatory response, such as CXCR3 and CCR5. Furthermore, some chemokine recruitment pathways are up-regulated relatively early in the inflammatory process and, because most clinical trials include patients with active disease refractory to existing medical therapy, the crucial role of this pathway may have been missed. Consistent with this hypothesis in both the phase II and phase III anti-CCR9 trials in Crohn’s, clinical remission rates were greater in the group who did not have previous or recent exposure to biologics.^38,54,69,70^

It is particularly important to consider heterogeneity in the IBD phenotypes recruited for clinical trials, and to take in to account clinical, genetic, and immunophenotypic factors that might define patient subsets most likely to respond. An obvious stratification in IBD is between Crohn’s disease and ulcerative colitis, but it may be equally important to take in to account the predominant site of disease, given the regional differences in the chemokines involved in immune cell recruitment between the small bowel and colon. For instance, in the small bowel the proportion of infiltrating CCR9^+^ T-cells remains relatively stable or may actually lessen as inflammation progresses,^39^ whereas in colitis CCL25 expression is increased in the colon and strongly correlates with inflammatory activity.^10^ The potential importance of this factor is emphasised by SHIELD-1 where differences between Vercirnon and placebo in CD were seen in patients with colitis but not small bowel disease.^54^ The Mongersen Phase II trial was considerably refined in light of these concerns.^92^ Given that the active compound is released in the terminal ileum and ascending colon, patients with lesions localised to the upper gastrointestinal tract or distal large bowel (beyond the transverse colon) were rightly excluded.^92,93^

Finally, we will increasingly use sophisticated, often unsupervised, data gleaned from genomics and deep immunophenotyping, to determine much more precisely the immune pathways activated in particular patients and to use this information to target patients for specific therapy—for instance, patients with IL-17-dominated responses may respond better to therapy targeted at CCR6 or CCL20. Current approaches are likely to be an oversimplification, and detailed probing of immune pathways using novel techniques in patients may allow a much more nuanced approach to therapy selection. Herein, IBD has a real advantage over many other diseases in that analysis can be carried out on the target tissue rather than having to be inferred from changes in blood. A recent study in rheumatoid arthritis shows the potential of such approaches. The investigators used multidimensional cytometry and single-cell transcriptomics to define a new population of helper T cells in the joints of some patients with active disease.^110^ These cells were associated with disease activity and expressed distinct combinations of chemokines and chemokine receptors. Thus, patients in whom these cells are shown to be present and activated could be targeted with specific combinations of anti-chemokine treatment aimed at preventing the recruitment of this specific cell type early in disease.

## Funding

PJT and DHA received institutional funding from the NIHR BRC. This article is independent research supported by the NIHR Birmingham Liver Biomedical Research Centre. The views expressed in this publication are of the authors and not necessarily those of the National Health Service, the NIHR, or the Department of Health.

## Conflict of Interest

There are no conflicts of interest to disclose.
