# Chemokines and Chemokine Receptors as Therapeutic Targets in Inflammatory Bowel Disease; Pitfalls and Promise

**DOI:** 10.1093/ecco-jcc/jjy130

**Published:** 2018-09-29

**Authors:** Palak J Trivedi, David H Adams

**Affiliations:** 1 National Institute for Health Research (NIHR) Birmingham Biomedical Research Centre (BRC), University of Birmingham and University Hospitals Birmingham NHS Foundation Trust, UK; 2 Institute of Immunology and Immunotherapy, University of Birmingham, Birmingham, UK; 3 Liver Unit, University Hospitals Birmingham NHS Foundation Trust, UK; 4 Department of Gastroenterology, University Hospitals Birmingham NHS Foundation Trust, UK

In the original article, the affiliations were incorrect and should have read as per the above.

The funding statement was also incorrect and should have read as follows.

## Funding

PJT and DHA received institutional funding from the NIHR BRC. This article is independent research supported by the NIHR Birmingham Liver Biomedical Research Centre. The views expressed in this publication are of the authors and not necessarily those of the National Health Service, the NIHR, or the Department of Health.

The authors apologize for the errors.

